# Quinazoline derivatives: synthesis and bioactivities

**DOI:** 10.1186/1752-153X-7-95

**Published:** 2013-06-03

**Authors:** Dan Wang, Feng Gao

**Affiliations:** 1 Department of Chinese Traditional Herbal, Agronomy College, Sichuan Agricultural University, No. 211, Huiming Road, Wenjiang Region, Chengdu, 611130, P. R. China

**Keywords:** Quinazoline derivative, Synthesis, Bioactivity

## Abstract

Owing to the significant biological activities, quinazoline derivatives have drawn more and more attention in the synthesis and bioactivities research. This review summarizes the recent advances in the synthesis and biological activities investigations of quinazoline derivatives. According to the main method the authors adopted in their research design, those synthetic methods were divided into five main classifications, including Aza-reaction, Microwave-assisted reaction, Metal-mediated reaction, Ultrasound-promoted reaction and Phase-transfer catalysis reaction. The biological activities of the synthesized quinazoline derivatives also are discussed.

## Introduction

Quinazoline derivatives, which belong to the N-containing heterocyclic compounds, have caused universal concerns due to their widely and distinct biopharmaceutical activities. Researchers have already determined many therapeutic activities of quinazoline derivatives, including anti-cancer [1-4], anti-inflammation [5,6], anti-bacterial [7-10], analgesia [5,9], anti-virus [11], anti-cytotoxin [12], anti-spasm [9,13], anti-tuberculosis [14], anti-oxidation [15], anti-malarial [16], anti-hypertension [17], anti-obesity [18], anti-psychotic [19], anti-diabetes [20], etc. Medicinal chemists synthesized a variety of quinazoline compounds with different biological activities by installing various active groups to the quinazoline moiety using developing synthetic methods. And the potential applications of the quinazoline derivatives in fields of biology, pesticides and medicine have also been explored. This review summarized the representative synthetic methods, either traditional or novel, and categorized them into five main classifications, including Aza-reaction, Microwave-assisted reaction, Metal-catalyzed reaction, Ultrasound-promoted reaction and Phase-transfer catalysis. Besides, three other kinds of reactions were also listed out, which were either designed as supplementary methods in most experiments or used as the main methods in some researches, including Oxidative cyclization, Reagent refluxing and One-pot synthesis. In addition, the bioactivity researches of quinazoline derivatives were also discussed in order to provide valuable reference for the future synthesis and biological investigation of these compounds.

## Review

### Synthetic methods

#### Aza-reaction

##### Aza-Diels-Alder reaction

**Scheme 1:**
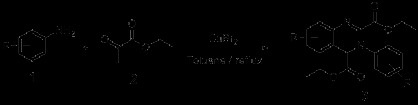
Synthesis of derivatives 3 by cascade imino-Diels-Alder reaction.

Imino-Diels-Alder reaction [21] containing the coupling of imine and electron-rich alkene gradually became a powerful tool for the synthesis of quinazoline derivatives [22]. In Povarov imino-Diels-Alder reaction, aniline and ethyl glyoxalate were chosen as substrates. And two molecules of *α*-iminoesters, which were got from the condensation of aniline and ethyl glyoxalate, were hypothesized to form the direct additive product. Cascade Imino-Diels-Alder reaction conducted by Chen *et al.*[23] was extended from the Povarov Imino-Diels-Alder reaction. In this research, researchers chosed the same substrates as in the Povarov Imino-Diels-Alder reaction, and adopted various kinds of Lewis acid as catalysts, then the reagents were refluxed in toluene for one day, and finally produced quinazoline derivatives 3. CuBr_2_ was determined as the optimized catalyst with highest yields (Scheme 1).

##### Aza-Wittig reaction

**Scheme 2:**
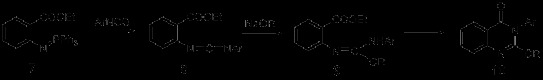
Synthesis of indolo[1,2-c]quinazolines 6 from azides 4.

**Scheme 3:**
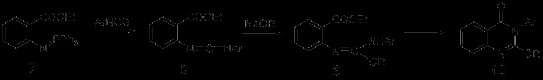
Synthesis of 2-alkoxy-3H-quinazolin-4-ones.

**Scheme 4:**
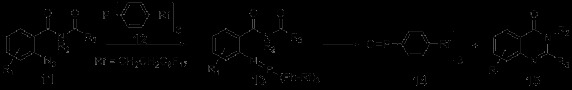
Synthesis of 3H-quinazolin-4-ones via aza-Wittig reaction.

Aza-Wittig reaction, which generally precedes in cascade with easy operation under mild reaction conditions, is widely used in the synthesis of N-heterocycles [24]. He *et al.* reported a kind of tandem Staudinger–Aza-Wittig–Nucleophilic addition reaction to synthesize indolo[1,2-c]quinazolines recently [25]. The main synthetic procedure of this research was using azides 4 and triphenylphosphine to react in toluene for 2 h at room temperature, and then heating at reflux for 6–24 h. Results showed that the nitrogen evolution through the Staudinger reaction halted during the initial 2 h, and surprisingly produced the final product indolo[1,2- c]quinazolines 6 directly from the reaction mixture (Scheme 2).A synthetic method for 2-alkoxy-3H-quinazolin-4-ones was reported by Ding *et al*. in 2004 [26]. In this study, twelve novel 2-alkoxy-3H-quinazolin-4-ones were synthesized from carbodiimide 8, which was obtained from aza-Wittig reaction of iminophosphorane 7 with aromatic isocynate (Scheme 3).Sophie Barthelemy *et al.* applied perfluoroalkyl-tagged triphenylphosphine in a fluorous biphasic system for the synthesis of 3H-quinazolin-4-ones by aza-Wittig reaction [27]. Compounds such as Type 11 were adopted as substrates to react quantitatively with per fluoro-tagged phosphine 12 to obtain iminophosphoranes 13. Then these intermediates converted directly into the desired quinazoline derivatives through intramolecular aza-Wittig reaction. The reactions were preceded in toluene as solvent and trifluorotoluene as co-solvent (Scheme 4). After the reaction, desired products 15 were separated through solid-extraction on fluorous reversed-phase silica gel, for that the unreacted iminophosphoranes 13 and phosphane oxide 14 would leave the perfluoro tag on silica gel, thus make the products be washed off effectively. In this way, it was made possible for simple segregation of quinazolin-4-ones even with unquantitative cyclization reaction.

### Microwave-assisted synthesis

Compared to traditional heating methods, microwave heating could expand reaction range as well as shorten the reaction time from a few days or hours to a few minutes. Thus, when applied in fields of organic synthesis, pharmaceutical chemistry and high-throughput chemistry, microwave heating shows greater advantage than traditional heating methods [28-31].

**Scheme 5:**
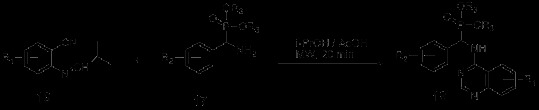
**Synthesis of quinazoline compounds containing α-aminophosphonat.**

Luo *et al.* reported the first microwave-assisted synthesis of new quinazoline derivates containing *α*-aminophosphonate [32]. In their method, N’-(substituted-2-cyanophenyl)-N,N-dimethyl-formamidine derivatives and dialkyl amino (phenyl) were adopted as the raw materials to react in 4:1 volume ratio of isopropanol to acetic acid solvent for 20 min under microwave irradiation (100°C, 100 psi), and obtained twenty-four quinazoline compounds 18, two of which had similar activity as commercial reagent Ningnanmycin (Scheme 5).

**Scheme 6:**
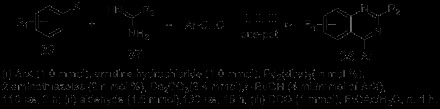
Microwave-assisted one-pot synthesis of quinazoline compounds.

Tu *et al*. reported a fast, one-pot, microwave-assisted synthesis of polysubstituent imidazo[1,2-a]quinoline, pyrimido[1,2-a]quinoline and quinolino[1,2-a]quinazoline derivatives [33]. They explored the optimal reagent, volume and heating temperature by testing different reagents under different reaction time and temperature. Then under the optimal conditions (2.0 mL glycol and 120°C),several aldehydes were separately reacted with various enaminones and malononitrile to obtain different products (Scheme 6).

**Scheme 7:**
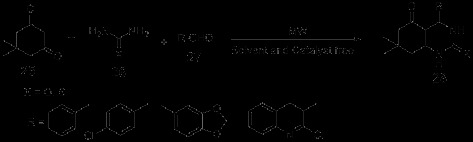
Solvent-catalyst-free microwave-assisted synthesis of quinazolines.

In the synthetic research conducted by Kidwai *et al.*[34], the target compounds quinazoline derivatives 28 were obtained by heating an equimolar amount of aldehyde 25, 5,5-dimethyl-1,3-cyclohexanedione (dimedone) 26 and urea/thiourea 27 under microwave irradiation in the absence of solvent and catalyst (Scheme 7).

Hazarkhani *et al.* adopted isatoic anhydride and 2-aminobenzimi-dazole as the raw materials, DMAC as solvent in their starting research and got 2-amino-N-(1-H -benzimidazol-2-yl) benzamide under microwave irradiation [35]. Then they discovered that this novel amide has three nucleophilic sites to condense with different electrophilic compounds, which made it applicable for the synthesis of numerous quinazolinone based targets, and was applied in the following synthesis.

**Scheme 8:**
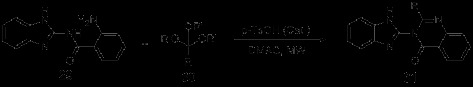
Synthesis of 3-benzimidazolyl-4(3H)-quinazolinones.

Amides (5 mmol), triethylorthoformate (7.5 mmol), p-tolue-nesulfonic acid (0.25 mmol) and DMAC (1–2 ml) were mixed in a tall beaker covered with stem-less funnel, and heated for 4 min under 600 w microwave power in a microwave oven, then interrupted in between with a cooling procedure. After that, the reaction mixture was cooled to room temperature and poured into a large volume of water for precipitation. Then the precipitate was filtered and recrystallized with 95% ethanol to obtain pure product 3-benzimidazolyl-4(3H)-quinazolinone (31) with a yield of 94% (Scheme 8).

### Metal-mediated reaction

#### Palladium-catalyzed reaction

Palladium-catalyzed coupling reaction, which plays a vital role in the pharmaceutical industry, is widely applied in chemical synthesis industry and laboratories as an efficient method for the formation of C-C and C-heteroatom bond.

**Scheme 9:**
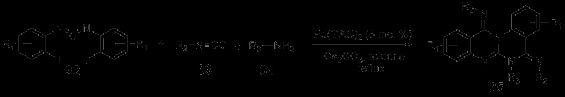
Synthesis of quinazolino[3,2-a]quinazolines.

Qiu *et al.* determined the optimum conditions for the palladium-catalyzed three-component synthesis of quinazolino[3,2-a]quinazolines as follows: amine (3.0 equiv), isocyanide (3.0 equiv), carbodiimide (0.2 mmol), Pd(OAc)_2_ (5 mol%) and Cs_2_CO_3_ (3.0 equiv) in 3.0 ml toluene (Scheme 9) [36].

**Scheme 10:**
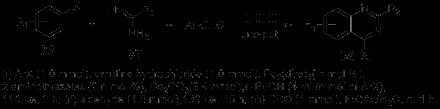
Palladium-catalyzed one-pot synthesis of quinazolines.

McGowan *et al.* developed a palladium-catalyzed one-pot synthesis of quinazoline derivatives [37]. The reaction process was shown in Scheme 10.

##### Zinc-reduced synthesis

**Scheme 11:**
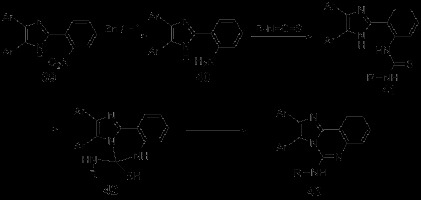
Synthesis of imidazo[1,2-c]quinazoline derivatives.

**Scheme 12:**
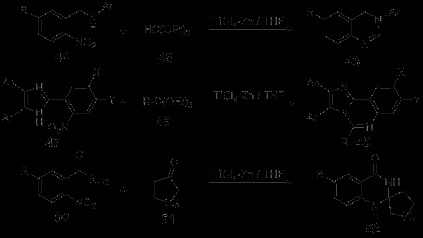
**TiCl**_**4**_**-Zn-mediated reduced synthesis of quinazoline derivatives.**

Zinc is the first capable metal found to participate in water-phase Barbier reaction. It could catalyze the allylation of carbonyl and carbonyl compounds as well as participate in the benzylation of carbonyl and some special alkylation. Apart from participating in the carbon-oxygen double bond Barbier reaction, Zinc could also be applied to carbon-nitrogen double bond Barbier reaction, such as the allylation of imine and *α*-amino aldehyde. In short, Zinc could stably exist in water phase with relatively strong activity. Active zinc obtained from ultrasonic-electrical method could even improve the reaction efficiency by more than three times. Although it often causes a few side effects, the cost-effectiveness and low-toxicity of zinc made it a good catalyst for organic reduction and synthetic reaction. In the synthetic research of imidazo[1,2-c]quinazoline derivatives designed by Shi *et al.*[38], 2-(2-nitro-phenyl)-1H-imidazoles 39 was reduced by Zn/H^+^ to 2-(2-aminop henyl)-1H-imidazoles 40, which then reacted with isothiocyanates to get intermediate 41. Cylization of compound 41 by nucleophilic attack of the nitrogen atoms on C = S group was afford the intermediates 42. Finally, the desired products 43 were obtained from 42 by losing of H_2_S (Scheme 11). Low-valent titanium reagents, which aroused an increasing concern in the field of organic synthesis, could effectively improve the coupling of carbonyl compounds [39]. A synthetic method assisted by low-valent titanium reagent was reported by the same group mentioned above [40]. In this synthesis, a series of quinazoline derivatives were afforded by adopting anhydrous THF as solvent and the TiCl_4_-Zn system as reducing agent. Several representative synthetic routes were selected, which were shown in Scheme 12.

##### Copper-catalyzed reaction

**Scheme 13:**
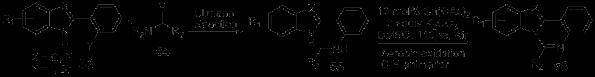
Copper-catalyzed synthesis of indolo[1,2-c]quinazoline derivatives.

**Scheme 14:**
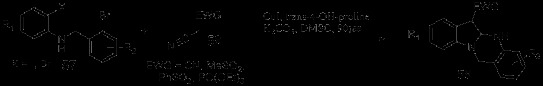
Copper-catalyzed one-pot synthesis of quinazolines derivatives.

Aryl ether, alkyl ether, aryl amine, alkyl amine, aryl sulfide, alkyl sulfide, etc., which are all very important structural fragments in many chemical molecules, have an urgent need for better synthetic methods. Classical copper-catalyzed Ullmann reaction has been widely studied due to its significant role in this regard. It raised attention from many chemists and became one of the focal point in organic chemistry research in recent years. Sang *et al.* reported a copper-catalyzed sequential Ullmann N-arylation and aerobic oxidative C-H amination for the convenient synthesis of indolo[1,2-c]quinazoline derivatives [41]. In their research, 2-(2-halophenyl)-1H-indoles and (aryl)methanamines were adopted as raw materials to generate corresponding Schiff base via Ullmann reaction. Then gas as oxidant, 3 equiv K_2_CO_3_ as base, DMSO as solvent and 10 mol% Cu(OAc)_2_ as catalyst were revealed as the optimum conditions, to conduct aerobic oxidative C-H amination under 110°C (Scheme 13).Jiang *et al.* also reported a copper-catalyzed one-pot synthesis of 5,12-dihydroindolo[2,1-b]quinazolines [42]. The best conditions of catalyst, ligand, base and solvent were determined as 10 mol% of CuI, 20 mol% of trans-4-hydroxyl-L-proline, 3.0 equiv of K_2_CO_3_, DMSO and 90°C, respectively. N-(2-bromobenzyl)-2-iodoani-line (57) and malononitrile (58) were adopted as the raw materials to afford desired compound 59 through copper-catalyzed intramolecular C-N coupling reaction (Scheme 14).

**Figure 1:**
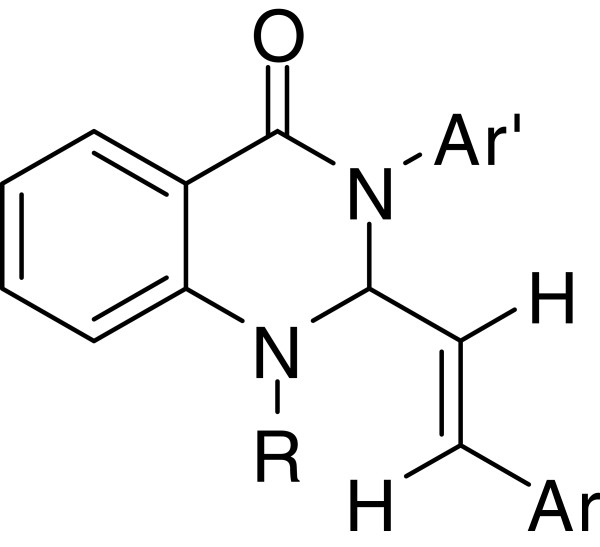
Structures of palladium-copper co-catalyzed synthesis of quinazolines.

A two-step catalytic synthesis of 2-substituted-1,2,3,4-tetrahydroquinazolinones was investigated by Kundu *et al.*[43], including the palladium-copper co-catalyzed C-arylation of terminal alkynes and copper-catalyzed cyclization of disubstituted alkynes. First of all, 2-(N-Alkyl-N-prop-2′-ynyl)amino-N’-p-tosyl benzamides and aryl iodides were adopted as the raw materials to react in presence of 5 equiv of Et_3_N for 16 h, catalyzed by 2.5 mol% (Ph_3_P)_2_PhCl_2_ and 5 mol% CuI, to generate a series of disubstituted alkynes. Then the products were cyclized in the presence of CuI (30 mol%), K_2_CO_3_ (2.5 equiv) and Bu_4_NBr (1 equiv) at 80°C for 16-24 h in CH_3_CN to afford the desired products 1-methyl(benzyl)-(E)-2-(2-arylvinyl)-3-p-tosyl-1,2,3,4-tetrahydroquinazoline-4-ones with high yield. The structures of obtained compounds were shown in Figure 1.

### Ultrasound-promoted synthesis

In critical synthesis, ultrasonic assistance is needed to meet the high requirements for temperature and pressure. For instance, in Bischler cyclization [44-46], the most traditional synthetic methods for quinazoline derivatives, high temperature (above 120°C) and high pressure are needed for at least 5 h in saturated ammonia alcohol solution. Various synthesis applying this method contains the passage of ammonia through a mixed melt of the amino compound and sodium acetate at a temperature higher than 160°C [47], in which ultrasonic promotion is demanded.

**Scheme 15:**
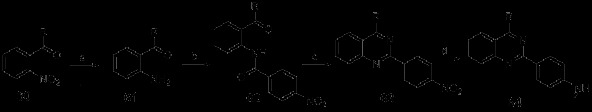
Ultrasound-assisted four-step synthesis of novel quinazoline derivatives.

Zhang *et al.* reported an ultrasound-assisted synthesis of novel quinazoline derivatives, including a four-step synthesis of quinazoline core and the optimization of the Bischler cyclization [48]. The optimum reagents and conditions of the four steps were as follows: (a) iron powder(reductant), concentrated HCl(catalyst), ethanol/water(co-solvents with V:V of 5:1), 50°C; (b) 4-nitrobenzoic acid chloride(1 equiv), TEA(1.2 equiv), DCM, 0°C; (c) 25% ammonia water, water, ultrasound 250 W, 80°C, 3 h; (d) iron powder, concentrated HCl, ethanol/water, 50°C (Scheme 15).

### Phase-transfer catalysis

Phase-transfer catalysis (PTC) is considered to be one of the promising methods in organic synthesis of specialty chemicals. The previous 20 years sees a steady increment in articles and patents dealing with PTC topics and their applications. Currently, rather than be simply used in replacement reactions, PTC is widely applied in polymer chemistry, heterocyclic chemistry, organometallic synthesis, agrochemicals, dyes, flavors, spices, and pharmaceutical technology [49-51].

**Scheme 16:**
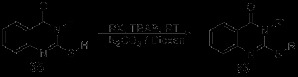
PTC synthesis of alkylation quinazoline derivatives.

In the synthetic research conducted by A. Kh. Khalil [52], the optimum conditions were determined as follows: Dioxane/anhydrous potasstium carbonate be set as liquid/solid phases and TBAB be set as catalyst. The 2-mercaptoquinazolin-4(3H)-one (65) was stirred effectively with haloorganic reagents under optimal conditions at 25°C for 2-4 h. Then in the following tests, compound 65 was treated respectively with ethyl bromide, allyl bromide, bromoactylacetone, and diethyl malonate bromide by molar ratio of 1:3, and afforded a series of quinazoline derivatives via S-monoalkylation. While treatments with several other compounds, including methyl iodide, benzyl bromide, *ω*-bromo-4-methoxyacetophenone, ethyl bromoacetate, and chloroacetyl chloride could afford products 66 via a simultaneous S- and N-dialkylation (Scheme 16).

**Scheme 17:**
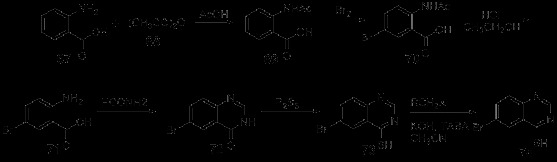
PTC synthesis of 4-alkylthio-quinazoline derivatives.

Yao *et al.* designed an investigation to bring bromine into the active structure of quinazoline sulfide [53]. Anthranilic acid was adopted as the starting material to generate a series of 6-bromo-4-alkylthioquinazoline compounds 74 via phase-transfer catalysis through a sequence of reaction, including acylation, bromination, hydrolysis, ring formation, vulcanization and thioether substitution (Scheme 17).

Apart from the five synthetic methods listed above, several other methods could also be used as main researching methods in some situation, while most of the time, they were set as auxiliary methods or necessary methods in experimental design. Here, several examples of such methods were listed.

### Oxidative cyclization

A three-step synthesis of mono- and bis-indolo[1,2-c]quinazolines was reported by Rohini *et al*. in 2010 [7]. In this research, the key indole precursor A was got from Fischer indole cyclization. And the corresponding intermediate mono and bis-2-(o-arylidineaminophenyl)indole, obtained from indole precursor A, then was put on oxidative cyclization with powdered KMnO_4_ in acetone to afford the desired products mono and bis-indolo[1,2-c]quinazoline.

In 2009, they also reported another synthesis of mono- and bis-6-arylbenzimidazo[1,2-c]quinazolines from corresponding 2-O-arylideneaminophenylbenzimidazoles by oxidative cyclization [54].

### Reagent refluxing

Chandrika *et al.* synthesized desired products from the intermediate obtained from reagent refluxing [12]. In the synthesis of tri-substituted products triazolo[4,3-a]-quinazolin-7-ones by Pandey *et al.*[55], the corresponding Schiff base was obtained from refluxing of key intermediate with isatin in methanol, which then cyclodehydrated to the products in concentrated sulfuric acid. Aside from these two researches, in some other synthetic researches [5,34,56], the intermediates or products were also obtained from refluxing of raw materials or intermediates in solvent.

### One-pot synthesis

In order to make the synthetic methods more convenient, many researchers gradually tend to integrate one-pot synthesis into their synthesis investigations. Such as microwave-assisted synthesis reported by Tu *et al.*[33], Copper-catalyzed domino synthesis reported by Jiang *et al.*[42], Palladium-catalyzed reaction reported by McGowan *et al.*[37] and Zinc-reduced synthesis reported by Shi *et al.*[38]. All of these reported methods were combined with one-pot synthesis.

### Bioactivity research

#### 4-position substituted quinazoline compounds

##### Melanin-concentrating hormone receptor 1 antagonists

**Figure 2:**
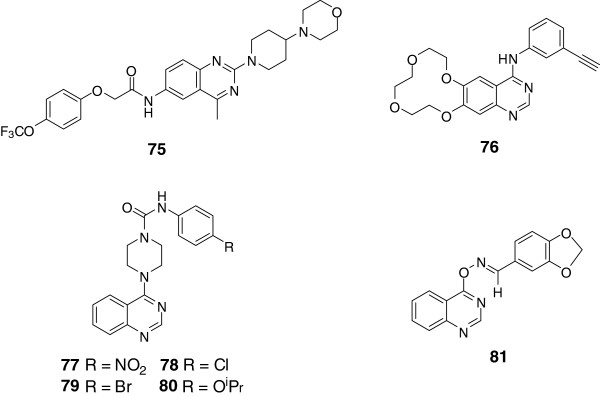
Structures of representative 4-substituted quinazolines with bioactivity.

MCHR1 antagonising quinazoline derivatives are proved to possess distinct anti-obesity activity. Sasmal *et al.* investigated the potential anti-obesity activity of quinazoline derivatives, which were determined as MCHR1 antagonists [18]. A series of compounds were obtained by the change of substituent groups, including 4-propyl-quinazolinone, 4-pyrrolidin-quinazolinone, 4-hydroxypiperidine-quinazoline, 4-pyrrolidin-quinazoline, 4-morpholinyl-quinazoline, etc. Firstly, the metabolic stability in blood and solubility of these compounds were studied. Then, their anti-obesity properties were tested. 4-Morpholinyl-quinazoline (75, Figure 2) showed good oral PK profile and was chosen as a prototype molecule used to test its effect in DIO C57BL/6 J mice. And the tested mice reached an obvious weight reduction of 12% in the fourteenth day, by oral administration of that compound (30 mg/kg, b.i.d.). The results showed, the representative compound 4-morpholinyl-quinazoline owned an obvious anti-obesity activity. However, it was also pointed out that the compound was in need of further improvement of stability in plasma related to the oxymethylene linker.Besides, there are a number of other quinazoline derivatives that also possess good inhibitory activity for MCHR1, including 4-amino-2-cyclohexyl aminoquinazoline, 4-dimethylamino quinazoline, etc. Among these compounds, which work as MCHR1 antagonists, some N-substituted amino quinazoline compounds exhibit high IC_50_ values due to their good affinity for human MCHR1, including ATC0065 and ATC0175 [57-59].

#### Epidermal growth factor receptor tyrosine kinase inhibitors

Researches suggest that EGFR tyrosine kinase inhibiting quinazoline derivatives possess significant anti-cancer activity. 4-Anilinoquinazoline showed a potent and highly selective inhibition for EGFR tyrosine kinase through ATP-competitive binding mechanism [60-66]. And quinazoline derivatives with aliphatic branch at 4-position of quinazoline core have moderate inhibitory activity for cyclin-dependent kinase [67].

Chandregowda *et al.* synthesized novel 4-anilinoquinazolines and evaluated their anti-cancer activity [1]. The new results indicated that quinazoline derivatives with alkyl-thiobenzothiazole side chain in 6-position and electron withdrawing group substituted in 4-aniline contain better biological activities.

Lately, synthesis and activity research on 4-anilinoquinazolines as well as extended researches on inhibitory activity of anilinoquinazolines for EGFR are reported continuously [68].For example, Hu *et al.* designed and synthesized several crown ether fused anilinoquinazoline analogues, combined with EGFR kinase in vitro test and EGFR mediated intracellular phosphorylation test [69]. IC_50_ values of these compounds range from 2 nM to 150 nM. The results indicated that substituent of ethynyl at meta-position or halogen on the 7-anilino were desirable for high potency. And among the products, compound 76 (Figure 2) expressed high resistance for EGFR and potent selectivity for kinases Abl and Arg, and showed good pharmacokinetic properties in preclinical evaluation. Moreover, it inhibited the growth of many human solid tumor xenografts in a dose-dependent way(range 50–100 mg/kg).

Acrolein amine quinazolines substituted on the 6-position could irreversibly bind with intracellular ATP binding domain of EGFR [70,71]. 6-Substituted-4-anilino quinazolines with irreversible binding property with EGFR were synthesized by Vasdev *et al.*, starting from [^18^ F]fluoroanilines [3].

There are some other researches aiming at replacing 4-anilino with other substituents [72-74], such as thiosemicarbazide, which possesses a variety of biological profiles, including anti-cancer, anti-fungi, anti-bacterial, anti-inflammation and anti-virus. Thus it acts as an efficient pharmacophore in drug design. In order to find novel quinazoline compounds with same enzyme sites as 4-anilinoquinazoline, a series of 4-thiosemicarbazide quinazolines were synthesized by He *et al.*[75], and their anti-cancer activities were estimated using 5 human cancer cell lines with 5-FU as reference. Preliminary results showed that some produced compounds exhibited better inhibitory activity against these 5 human cancer cell lines than 5-FU. Structure-activity relationship results indicated that compounds with unsubstituted quinazoline ring and benzene ring or chloro/fluoro substituted benzene ring were proved to have higher anti-cancer activity.

### Platelet-derived growth factor receptor phosphorylation inhibitors

Cell proliferation induced by unusual platelet-derived growth factor receptor (PDGFR) will lead to a variety of proliferative diseases such as atherosclerosis, restenosis following PTCA, glomerulonephritis, glomerulosclerosis, liver cirrhosis, pulmonary fibrosis, and cancer [76-86]. PDGFR phosphorylation inhibitors are potential treatments for these proliferative diseases [87].

Matsuno *et al.* obtained a series of compounds containing 4-piperazinyl substituted quinazoline core from screening of PDGFR phosphorylation inhibitors [87]. KN1022 was adopted as the prototype inhibitor in structure-activity relationship analysis, and the 4-nitrobenzene urea moiety was studied, which indicated that compounds with substitution on 4-position of benzene ring worked best, but activity would be reduced with more than two substituents on benzene ring. In their research, several KN1022 (77) (Figure 2) derivatives with favorable activity were obtained, including 4-(4-methylphenoxy)phenyl,4-tert-butylphenyl and 4-phenoxyphenyl. In vivo assays were conducted by oral administration of these three compounds (30 mg/kg, twice daily) to SD rats. 4-chlorophenyl (78), 4-bromophenyl (79), and 4-isopropoxyphenyl (80) (Figure 2) analogues were then proved to have obvious inhibitory activity against neointima formation in the carotid artery of the balloon catheter de-endothelialized vessel in the rats.

### Germicide

Li *et al.* synthesized and biologically evaluated a series of 4-quinazoline oxime ether compounds in purpose of discovering novel acaricides [11]. Compounds obtained in this research were proved to have different degrees of suppression for phytovirus TMV, among which compound 81 (Figure 2) showed potent in vivo and in vitro activity against TMV, 65% and 61% respectively. What’s more, bioassays showed that compound 81 also exhibited favorable inhibitory activities against CMV,PVX and PVY after virus vaccination.

#### Heterocyclic quinazoline derivatives

##### Imidazo-quinazolines

**Figure 3:**
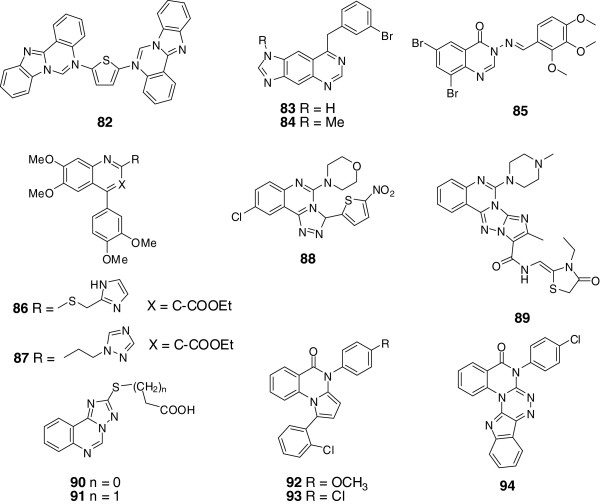
Structures of representative heterocyclic quinazoline derivatives.

In the synthesis of mono and bis-6-arylbenzimidazo[1,2-c]quinazolines by Rohini *et al.*[54], bioassays were conducted applying standard drugs Ampicillin and Ketoconazole as references. Among the products, the MIC of compound 82 (Figure 3) against Staphylococcus aureus, bacillus subtilis, streptococcus pneumoniae, Salmonella typhimurium, escherichia coli, klebsiella pneumoniae, aspergillus niger, candida albicans and Trichoderma viridae were 2.5, 10, 5, 5, 2.5, 5, 2.5, 2.5, 5 μg/mL respectively.Rewcastle *et al.* synthesized several fused tricyclic quinazoline analogues and investigated their enzyme inhibitory activity [88]. Linear imidazo[4,5-g]quinazoline (83) was proved to be the most potent compound with an inhibition IC_50_ of 0.008 nM, and the N-methyl analog (84) showed similar activity as compound 83 (Figure 3). In the imidazo[4,5-g]quinazoline and pyrroloquinazoline series, the angular isomers showed much weaker inhibitory ability than the linear compounds, which was consistent with the results of the previous structure-activity relationship. Meanwhile, small electron-donating substituent at the 6- or (and) 7-positions was beneficial to the inhibitory activity.

##### Acylhydrazone quinazolines

Acylhydrazone is a pharmacophore armed with favorable activity of antibiosis, anticancer and antivirus. Panneerselvam *et al.* synthesized several acylhydrazone quinazolines via condensation reaction of 3-amino-6, 8-dibromo-2-phenyl-quinazolin-4(3H)-ones and aromatic aldehyde [13]. Among which, the representative compound 85 (Figure 3) showed the most significant activity against *S. aureus*, *S. epidermidi*, *M. luteus*, *B. cereus*, *E. coli*, *P. aeruginosa*, *K. pneumoniae*, *A. niger* and *A. fumigatus*.

##### Triazole quinazolines

Triazole quinazolines aroused attention for their various bioactivities. Baba *et al.* conducted anti-inflammatory research on quinazoline derivatives [6]. Justicidins, the potent bone resorption inhibitors, were adopted as lead compounds, and quinazoline derivative 86 was discovered to show favorable anti-inflammatory effect on rats with adjuvant arthritis. Then the structure of compound 86 was modified by adding heteroaryl moiety on the alkyl side chain at 2-position of the skeleton, result of which showed that installation of an imidazole or a triazole moiety on the 2-alkyl side chain could increase the anti-inflammatory. Then, compound 86 with ED_50_ of 2.6 mg/kg/day was selected as candidate for further research, which showed that the inhibitory ability against Th1 cytokine production of 87 was considered to be its significant immune regulating function (Figure 3).Fifteen [1,2,4] triazole [4,3-c] quinazoline derivates were synthesized and evaluated for their antimicrobial activity by Jantova *et al.*[89], among which, compound 88 (Figure 3) was found with the highest potency against *Bacillus subtilis*, *Staphylococcus aureus*, *Candida tropicalis* and *Rick-ettsia nigricans*.Nasr *et al.* evaluated the antimicrobial activity of novel 1,2,4-triazolo[4,3-c]-quinazoline analogues against typical gram-positive bacterium and Gram-negative bacterium [90]. Among these analogues, tetracyclic compound 89 showed higher activity than the reference drug of ciprofloxacin (Figure 3).Besides, 2-thio-[1,2,4]triazolo[1,5-c]quinazolinones with C-5 substituted by sulfo-alkyl groups were discovered with moderate antimicrobial activity [91-93]. Based on the former researches, Antipenko *et al.* synthesized novel 2-thio-[1,2,4]triazolo[1,5-c]quinazoline derivatives and investigated their bioactivities [8]. *Escherichia coli*, *Pseudomonas aeruginosa*, *As-pergillus niger*, *Mycobacterium luteum*, *Candida albicans* and *Candida tenuis* were applied in the antimicrobial test, which showed that compounds 90 and 91 exhibited obvious suppression for *Candida albicans*, which was validated by further bioluminescence inhibition test and related to their lipophilicity (Figure 3).Pandey *et al.* also conducted antimicrobial researches on novel quinazolinones fused with [1,2,4]-triazole, [1,2,4]-triazine and [1,2,4,5]-tetrazine rings [55]. Among the quinazolinones derivates, compounds 92, 93 and 94 (Figure 3) showed excellent activities against *Escherichia coli*, *pseudomonas aeruginosa*, *streptococcus pneumoniae*, and *bacillus subtilis*.

### Other quinazoline analogues

#### 2,3-Disubstituted quinazolines

**Figure 4:**
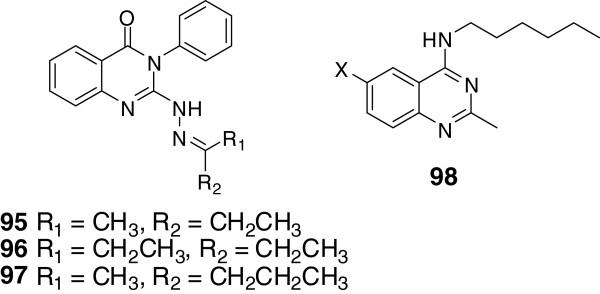
Structures of representative other quinazoline analogues.

2,3-Disubstituted quinazolin-4(3H)-ones have been discovered with favorable analgesic and anti-inflammatory function [94,95]. Alagarsamy *et al.* reported several 2,3-disubstituted quinazoline analogues with potent analgesic and anti-inflammatory activity, such as 2-phenyl-3-substituted quinazolines [96], 2-methyl-3-substituted quinazolines [97], 2-methylthio-3-substituted quinazolines [98] and 2,3-disubstituted quinazolines [99]. On the basis of these researches, they synthesized novel 3-phenyl-2-substituted-3H-quinazoline-4-ones in purpose of further reducing the ulceration side effects [5]. And the analgesic, anti-inflammatory and ulcerogenic index activities of these compounds were tested. Among the synthesized derivates, compounds 95, 96 and 97 (Figure 4) showed moderate analgesic activity. It is worth to mention that compound 97 exhibited higher anti-inflammatory potency, reference to standard drug of diclofenac sodium. In addition, the evaluated compounds all caused milder ulceration side effects, reference to aspirin.

#### Indole-involved quinazolines

Indole moiety involved heterocycles are proved to have a wide variety of pharmaceutical and medical profiles, such as anti-inflammation, anti-microbial, anti-cancer, anti-malarial, etc. [100-106]. Rohini *et al.* conducted anti-microbial study on the newly produced 6-substituted indolo[1,2-c] quinazolines applying gram-positive bacterium (*Staphylococcus aureus*, *Bacillus subtilis* and *Streptococcus pyogenes*), gram-negative bacterium (*Salmonella typhimurium*, *Escherichia coli* and *Klebsiella pneumonia*), and pathogenic fungus (*Aspergillus niger*, *Candida albicans* and *Trichoderma viridae*) as the test bacterium, and standard drug ampicillin and ketoconazole as reference, which indicated that some of the synthesized compounds showed favorable inhibition against the tested microorganism [7].

#### 2,4,6-trisubstituted quinazolines

Chandrika *et al.* synthesized and biologically evaluated various 2,4,6-trisubstituted quinazoline derivatives. The core of compound 98 (Figure 4) showed antimicrobial activities against gram-positive bacterium and gram-negative bacterium. In addition, it could be drawn from the SAR that decylamine group substituted at C-4 is beneficial to the activity while iodo-group at C-6 is detrimental to activity [12].

## Conclusions

Traditional synthetic methods for quinazoline derivatives, still in general use, including Aza-synthetic method, refluxing, oxidative cyclization, are fundamental methods for the synthesis of this important heterocyclic compounds. It could be seen from the examples compiled above that some novel synthetic methods are in constant development, and different methods are adopted in the synthesis of different quinazoline analogues, such as phase-transfer synthesis, ultrasound-promoted synthesis, etc. The gradually improved synthetic methods better the synthetic research on quinazoline derivatives with a tendency of faster, more diverse and more convenient. Then, for another, it is known that substituents at different positions affect the activity differently. For instance, quinazoline derivatives with imidazole substituted at the 2-position of side chain own potent anti-inflammatory function; and quinazoline derivatives with amine or substituted amine on 4-position and either halogens or electron rich substituent groups on 6-position could promote the anti-cancer and anti-microbial activities [7,12], etc. By careful observation of the recent researches, 2-, 4- and 6-position substituted quinazoline analogs remain majority among the products. However, with the deepening and development of researches, substituent groups at other positions are also achieved and studied increasingly, such as the construction of N-heterocyclic quinazolines by introduction of active groups into 3-position of quinazoline core. It is worth mentioning that N-heterocyclic quinazolines with more rigid and complicated structure were synthesized successively, some of which showed excellent antimicrobial properties. In addition, it could be drawn from the research progress above that enhancement of activity by splicing method of installing various active groups is and will still be the main method for drug design and reconstruction of quinazoline derivatives.

## Competing interests

The authors declare that they have no competing interests.

## Authors’ contributions

FG and DW have been involved in preparing the manuscript. The two authors are thought to have equal contributions. Both authors have read and approved the final manuscript.
